# Genetic and Environmental Contributions to Subcortical Gray Matter Microstructure and Volume in the Developing Brain

**DOI:** 10.1007/s10519-023-10142-1

**Published:** 2023-04-26

**Authors:** Richard Watts, Lydia Rader, Justin Grant, Christopher G. Filippi

**Affiliations:** 1 Department of Psychology, Yale University, 2 Hillhouse Avenue, New Haven, CT 06520 USA; 2 Institute for Behavioral Genetics, Department of Psychology and Neuroscience, University of Colorado Boulder, Boulder, CO USA; 3 Department of Radiology, Tufts University School of Medicine, Boston, MA USA

**Keywords:** Genetics, Deep gray matter, Diffusion MRI, Development

## Abstract

Using baseline (ages 9–10) and two-year follow-up (ages 11–12) data from monozygotic and dizygotic twins enrolled in the longitudinal Adolescent Brain Cognitive Development^SM^ Study, we investigated the genetic and environmental contributions to microstructure and volume of nine subcortical gray matter regions. Microstructure was assessed using diffusion MRI data analyzed using restriction spectrum imaging (RSI) and diffusion tensor imaging (DTI) models. The highest heritability estimates (estimate [95% confidence interval]) for microstructure were found using the RSI model in the pallidum (baseline: 0.859 [0.818, 0.889], follow-up: 0.835 [0.787, 0.871]), putamen (baseline: 0.859 [0.819, 0.889], follow-up: 0.874 [0.838, 0.902]), and thalamus (baseline: 0.855 [0.814, 0.887], follow-up: 0.819 [0.769, 0.857]). For volumes the corresponding regions were the caudate (baseline: 0.831 [0.688, 0.992], follow-up: 0.848 [0.701, 1.011]) and putamen (baseline: 0.906 [0.875, 0.914], follow-up: 0.906 [0.885, 0.923]). The subcortical regions displayed high genetic stability (rA = 0.743–1.000) across time and exhibited unique environmental correlations (rE = 0.194–0.610). Individual differences in both gray matter microstructure and volumes can be largely explained by additive genetic effects in this sample.

**Supplementary Information:**

The online version contains supplementary material available at 10.1007/s10519-023-10142-1.

## Introduction

Understanding how genetic variability influences brain structure in adolescence may provide insights into the pathophysiology and biological constructs that impact normal brain development, cognition, and the development of neuropsychiatric disorders. Brain structures vary in heritability, suggesting that different genes may influence the structural development of different areas (Bis et al. 2012; den Braber et al. 2013; Stein et al. 2012) and the genetic influences may vary over time (Brouwer et al. 2017). Subcortical regions of the brain are associated with neuropsychiatric disorders including depression (Koolschijn et al. 2009), anxiety (Holzschneider and Mulert 2011), and schizophrenia (Shepherd et al. 2012), as well as intellectual functioning (Bohlken et al. 2014).

Studies, predominantly in adult populations, have investigated the heritability of subcortical volumes of anatomic structures in the brain (den Braber et al. 2013; Kremen et al. 2010). Hippocampal and intracranial volume have been associated with genes located on chromosome 12q24 (Bis et al. 2012; Stein et al. 2012; Yoon et al. 2011). A meta-analysis of twin studies reported high heritability from 0.52 for the right thalamus to 0.82 for the right putamen volume, but with a relatively small sample size and wide confidence intervals of 0.4–0.8 for the right thalamus (Blokland et al. 2012). Another twin study that looked at subcortical volumes over a 5 year period also found high heritability for the thalamus, caudate, and putamen (den Braber et al. 2013). Other studies on adult twins have demonstrated high heritability of total intracranial brain volume (Posthuma et al. 2000; Thompson et al. 2001).

The brain undergoes rapid development in the first two years of life and nears adult size by early childhood at around age six (Jansen et al. 2015), but more dynamic changes in cortical and subcortical regions involving gray and white matter continue to occur into adolescence and young adulthood (Lenroot and Giedd 2006). Prior work in neurodevelopment has suggested greater genetic heritability earlier in childhood for primary cortices (motor, somatosensory), and more heritability in later adolescence for structures unique to the human species such as the dorsolateral prefrontal cortex (Lenroot and Giedd 2006).

There are few reports on adolescents with respect to heritability of subcortical volumes. A study of twins scanned at ages 9 and 12 showed high heritability for intracranial volume, gray matter, and white matter volume from 0.77 to 0.91 (Peper et al. 2009). In another study of 8 year-olds, heritability ranged from 0.57 to 0.79 for gray matter, white matter, corpus callosum, and total cerebrum, but with higher heritability in the left cerebral hemisphere (Yoon et al. 2011). A larger study of 326 twins resulted in heritability measures of 0.65 for corpus callosum, 0.64 for basal ganglia, and 0.42 for the thalamus, but lower for the ventricles (0.17) and cerebellum (0.24) (Schmitt et al. 2007).

Phenotypic neurodevelopmental trajectory research shows that there are significant patterns of age-related change in subcortical volumes across adolescence (Herting et al. 2018); however, it is unclear which etiological influences underlying the phenotypes remain stable. Longitudinal twin research allows for testing whether the genetic and environmental influences on subcortical structure are stable in adolescence, or if there are new genetic influences that emerge. Anomalies in brain structure are a robust correlate to adolescent and adult psychopathology (Gurholt et al. 2022; Guyer 2020), and understanding the stable and emerging influences in brain development may provide insights into the relationship between genetics, brain development and mental health.

One previous longitudinal twin study found that subcortical volumes (thalamus, hippocampus, amygdala, putamen, caudate, pallidum, and nucleus accumbens) were highly heritable and entirely genetically stable from ages 9 to 12; however the sample size was limited (48 MZ pairs; 64 DZ pairs)(Swagerman et al. 2014).

### ABCD study

While several studies have considered the heritability of the volumes of subcortical structures, very few have investigated their microstructure (Gillespie et al. 2017), which can be probed non-invasively using diffusion MRI. In this study, we leverage the Adolescent Brain Cognitive Development^SM^ Study (ABCD Study^→^) to investigate the heritability of both subcortical volume and microstructure in adolescents.

The ABCD study is a longitudinal study of brain development that includes extensive cognitive, behavioral, genetic and imaging data (Casey et al. 2018). The study is enriched for same sex twin pairs (Iacono et al. 2018), enabling the assessment of the genetic and environmental influences on a wide variety of traits over development. We use twin data to estimate the genetic and environmental influences on deep gray matter volume, assessed using conventional structural MRI, and microstructure, assessed using diffusion MRI.

The imaging battery included in ABCD, and repeated every two years, includes structural and diffusion MRI. Structural MRI scans have high (1 mm) spatial resolution, and sufficient gray/white matter contrast to enable segmentation of deep gray matter structures, and determination of their volumes. Diffusion MRI uses the motion of water molecules in the brain to provide information on the microscopic environment, and is sensitive to developmental changes related to, for example, myelination and cell density (Watts et al. 2003).

To our knowledge, this is the largest twin sample used to assess subcortical volume and microstructure heritability, as well as the first to longitudinally test the genetic and environmental contributions to subcortical microstructure in adolescence.

## Data and methods

### Subject selection

The ABCD data used in this report came from Curated Data Release 4.0. To maximize data consistency for the technically demanding diffusion acquisition, only data from twins scanned using a single MRI vendor (Siemens Healthineers AG) were included in the diffusion analysis. To maximize sample size, volumetric data for twins scanned on both Siemens and Philips (Philips HealthTech) scanners was included. Twin pairs were excluded if they had incomplete data, or if the structural and diffusion imaging data did not pass ABCD-recommended quality control, including a diffusion mean motion threshold of 1.5 mm. Subjects with a history of brain injury, cerebral palsy, muscular dystrophy, multiple sclerosis, or substance abuse were excluded. Demographic information for the two groups is given in Table 1.

**Table 1:** Demographics of participants (number of same-sex twin pairs) recruited and included in structural and diffusion analyses

	Recruited		Inclusion + Structural MRI QC (Siemens + Philips)		Diffusion MRI QC (Siemens only)	
Visit	BL	Yr2	BL	Yr2	BL	Yr2
Number of individuals	1535	1391	1407	1031	902	715
Monozygotic twin pairs	333	298	284	207	148	134
Same-Sex dizygotic twin pairs	432	393	369	247	212	158
Sex						
Female	378	345	315	215	175	136
Male	387	346	338	239	185	156
Race/ethnicity					p = 0.016	
White	507	464	436	306	**263**	204
Black	109	96	84	64	**26**	27
Hispanic	75	67	67	43	**36**	33
Asian	1	1	1	0	**0**	0
Other	73	63	65	41	**35**	28
Income					p = 0.040	
< $50k	122	108	104	72	**45**	46
$50k - $100k	215	197	184	137	**104**	82
> $100k	386	349	332	220	**202**	151
N/A	42	37	33	25	**9**	13
Parental education						
No HS diploma	12	12	10	8	6	5
HS diploma/GED	37	31	25	21	7	9
Some college	199	175	170	124	86	82
Bachelor	252	229	222	153	137	107
Post graduate	265	244	226	148	124	89

Bold font indicates measures that are significantly different from the as-recruited baseline sample (chi-squared test, p < 0.05)

### MRI data acquisition

The MRI acquisition is described in more detail elsewhere (Casey et al. 2018). Briefly, structural images were acquired using a T1-weighted 3D MP-RAGE sequence with 1 mm isotropic spatial resolution, TE/TI/TR = 2.88/1060/2500ms, flip angle = 8°, sagittal field of view of 256 × 256 × 176 mm^3^. Real-time motion detection and correction using volumetric navigators was used to minimize the effects of head motion (Tisdall et al. 2012).

Diffusion-weighted images were acquired using a 2D spin-echo EPI sequence with b-values of 500 (6 directions), 1000 (15 directions), 2000 (15 directions), and 3000 (60 directions) s/mm^2^, as well as 6 reference acquisitions without diffusion weighting. Diffusion imaging was acquired with 1.7 mm isotropic spatial resolution, TE/TR = 88/4100ms, 81 slices with a multiband factor of 3, axial field of view of 240 × 240 mm^2^. Corresponding field maps were acquired to enable correction of EPI susceptibility distortions.

### MRI data processing

#### Volumetric data

Imaging data was preprocessed as described previously (Hagler et al. 2019). Segmentation of subcortical structures was performed using FreeSurfer (Fischl et al. 2002), providing measures of the volume of each structure and masks for the aligned diffusion data. Subcortical volumes were normalized to the total intracranial volume.

#### Restriction spectrum imaging

A restriction spectrum imaging (RSI) (White et al. 2013) model was applied to the diffusion data to generate measures of the isotropic and directional restricted and hindered components of diffusion, as well as a free water component. The isotropic restricted component has been shown to be sensitive to tissue cellularity (White et al. 2013). Deep gray matter structures display low directional anisotropy, so we limited our analysis to the restricted normalized isotropic (RNI) component.

#### Diffusion tensor imaging

A conventional diffusion tensor imaging (DTI) model was also applied to generate measures of mean diffusivity (MD) to compare its sensitivity to the more sophisticated RSI model.

Zygosity status was genetically determined. All data was residualized to account for the effects of age, sex, and site.

#### Descriptive statistics

Intraclass correlations (ICCs) between MZ and DZ twin pairs were calculated as descriptive statistics to visualize the relative twin similarity for the phenotypes. Within subject Pearson correlations for phenotypes derived at baseline and year 2 were calculated to provide a proxy measure of the test-retest reliability of the imaging metrics. The relationship between the estimated heritability for each region/metric and the within-subject stability was calculated as a Pearson correlation.

#### Assumptions of the ACE model

The analysis presented is based on the ACE model. MZ twins share 100% of their genetic information, while DZ twins share approximately 50%. Additive genetic effects imply that the expected genetically-based correlation between DZ twins should be half that of MZ twins. Non-linear effects, such as those caused by genetic dominance, are not included in the model. The ACE model also incorporates the equal environments assumption (EEA), that the effect of the common environment is the same for MZ and DZ twins. Violation of this assumption would be expected to result in greater similarity of MZ twins relative to DZ twins, increasing the apparent heritability. The unique environment term models uncorrelated differences between twin pairs, including differences caused by measurement error.

#### Structural equation modeling

Structural Equation Modelling was used to assess the additive genetic (A), common environment (C), and unique environmental (E) contributions to tissue microstructure using the OpenMx (Boker et al. 2011) package in R (R version 3.6.1, R Core Team). A direct variance ACE model was used (Verhulst et al. 2019). Correlated factors models were employed to assess the stability of the variance components by decomposing the cross-time covariance of the trait into 3 latent sources of contributions (rA, rC, or rE)(Loehlin 1996). Likelihood-based confidence intervals are provided to account for the non-normal distribution of the variance components (Neale and Miller 1997). If the estimates’ confidence interval does not include 1.00, this is suggestive of novel etiological influences. Model fit was assessed using the criteria of root-mean-square error (RMSEA) < 0.06 and a Comparative Fit Index (CFI) > 0.95 (Hu and Bentler 1998). Chi-square difference tests were used to assess significance (p < 0.05) of the etiological correlations to account for the standard error invariance to model parameterization in genetic models (Neale et al. 1989).

## Results

### Subjects

The demographics of participants recruited and included in structural and diffusion analyses are shown in Table 1. For the volumetric measures at baseline, 284 MZ and 369 DZ twin pairs had acceptable quality data and were included in the analysis. For diffusion analysis, 148 MZ and 212 DZ twin pairs were included at baseline. At two-year follow-up the corresponding numbers were 207/247 and 134/158 for volume and diffusion respectively. The MZ and DZ groups did not differ significantly in age, sex, household income, parental education, or race/ethnicity (chi-squared test, p > 0.05).

### Baseline results

**Fig. 1:**
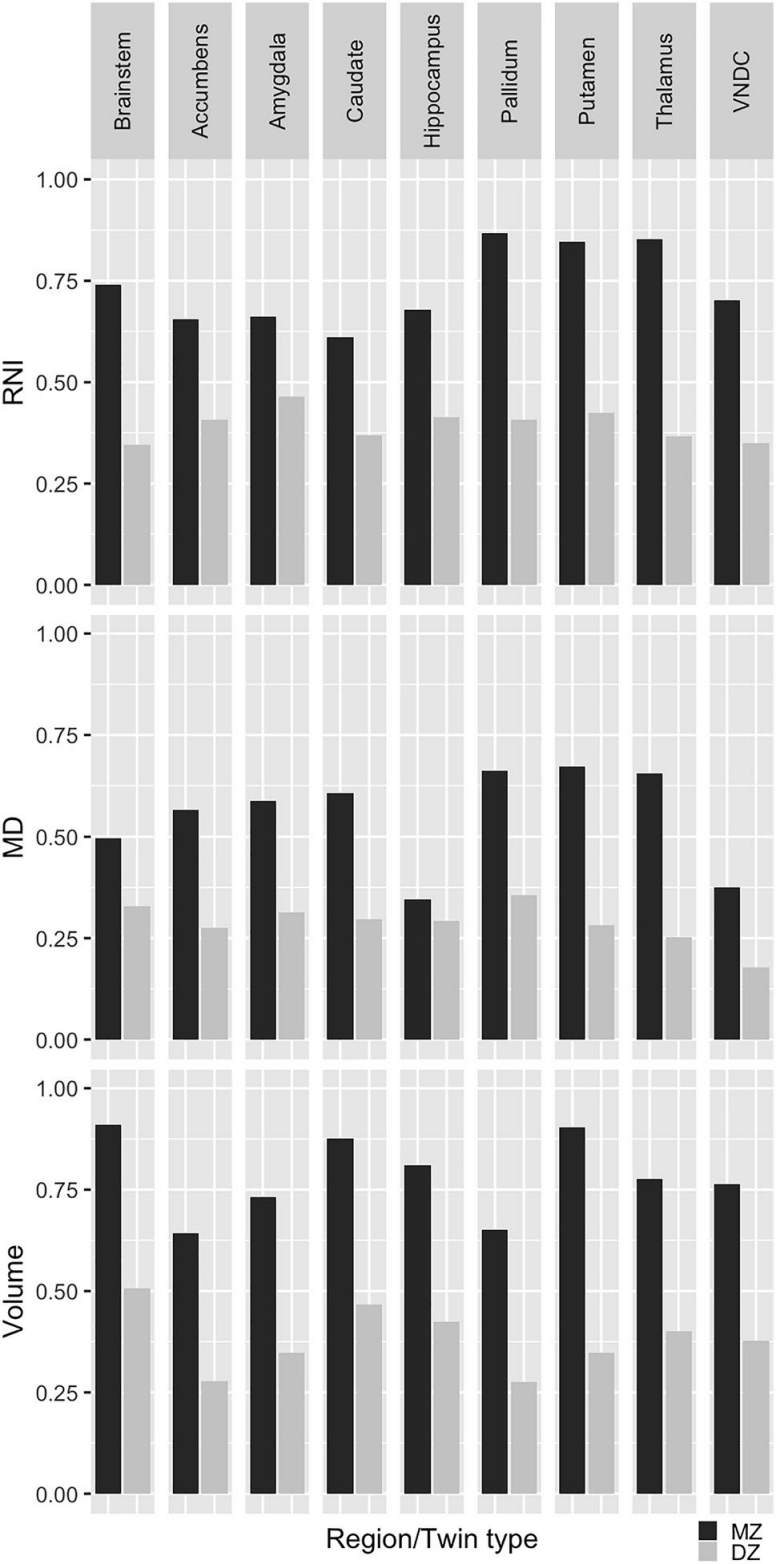
Intraclass correlation (ICC) between monozygotic (MZ) and dizygotic (DZ) twins for restricted normalized isotropic (RNI, top) signal, mean diffusivity (MD, middle), and volumes (bottom) of subcortical regions at baseline

Intraclass correlations (ICCs) for MZ and DZ twin pairs for each region and metric at baseline are shown in Fig. 1. For MZ twins, ICCs ranged from 0.610 (caudate) to 0.867 (pallidum) for RNI, 0.347 (hippocampus) to 0.673 (putamen) for MD, and 0.642 (nucleus accumbens) to 0.910 (brain stem) for volumes. With the exception of MD in the hippocampus and brainstem, the ICCs for DZ twins were significantly less than those from MZ twins (two sample t-test, p < 0.05).

**Fig. 2:**
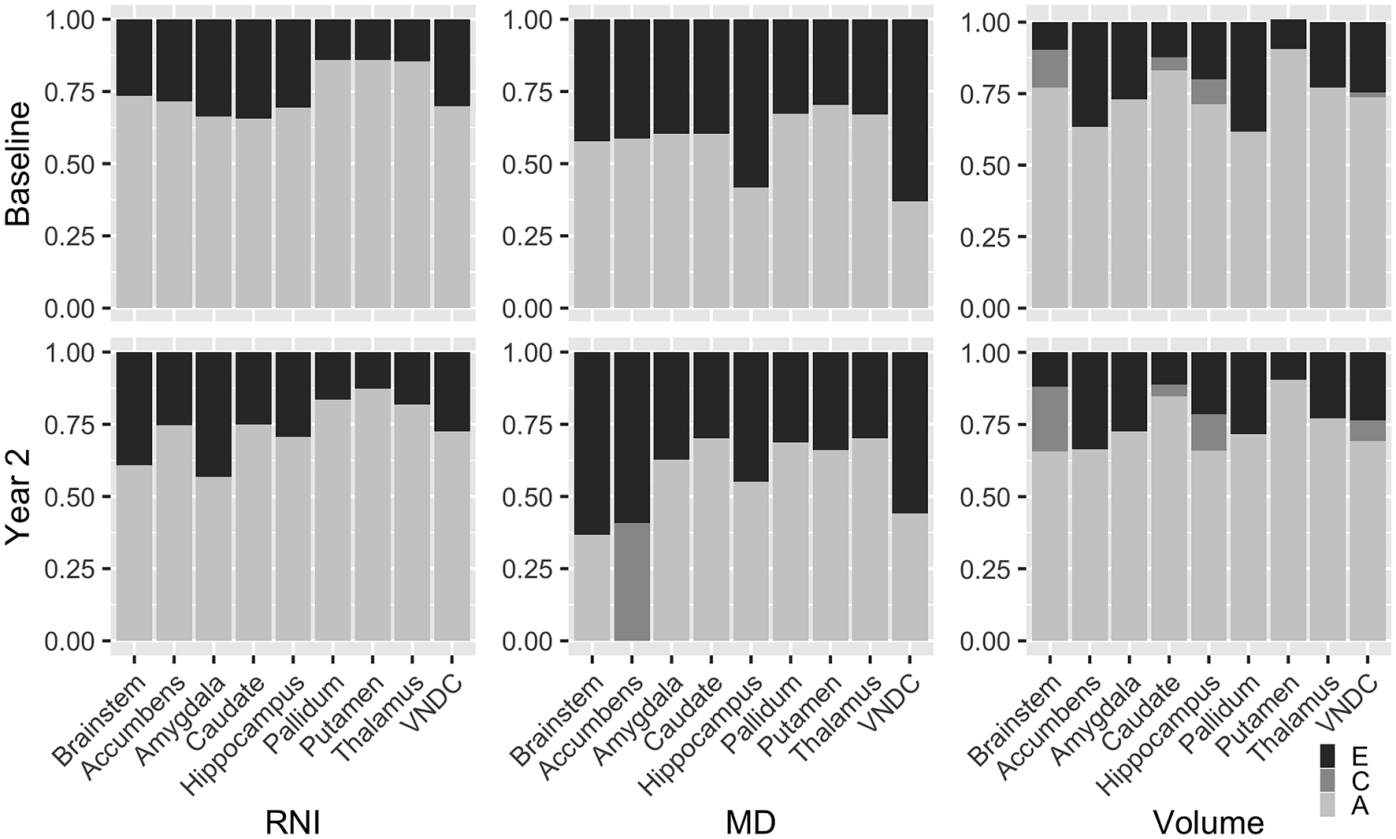
Additive genetic (A), common environmental (C), and unique environmental contributions to restricted normalized isotropic (RNI, left) signal, mean diffusivity (MD, middle), and volumes (right) in subcortical regions at baseline (top) and two-year follow-up (bottom). Negative estimates for contributions to MD in nucleus accumbens have been replaced with zeros for display

Structural equation modelling estimates of the additive genetic, common, and unique environmental contributions to each region and metric are shown in Table 2; Fig. 2 (top). The model fit statistics can be found in Supplementary Tables 1–3. Heritability estimates (estimate, 95% confidence interval) for RNI at baseline in the pallidum (0.859, 0.818–0.889), putamen (0.859, 0.819–0.889), and thalamus (0.855, 0.814–0.887) were especially high. For MD, heritability was generally lower than for RNI, but highest in the putamen (0.704, 0.622–0.768), pallidum (0.672, 0.596–0.734), and thalamus (0.670, 0.573–0.744). For structure volumes, the putamen (0.906, 0.875–0.914) and caudate (0.831, 0.688–0.992) were found to be highly heritable.

**Table 2:** Univariate variance component estimates from the correlated factor models

Measure	Region	A Baseline	A Year 2	C Baseline	C Year 2	E Baseline	E Year 2
RNI	Brainstem	0.736 [0.661, 0.793]	0.609 [0.510, 0.691]	–	–	0.264 [0.207, 0.339]	0.391 [0.309, 0.490]
	Accumbens	0.715 [0.644, 0.772]	0.747 [0.673, 0.804]	–	–	0.285 [0.228, 0.356]	0.252 [0.196, 0.327]
	Amygdala	0.663 [0.580, 0.730]	0.567 [0.460, 0.654]	–	–	0.337 [0.270, 0.420]	0.433 [0.346, 0.540]
	Caudate	0.655 [0.574, 0.722]	0.749 [0.677, 0.804]	–	–	0.345 [0.278, 0.426]	0.251 [0.196, 0.323]
	Hippocampus	0.695 [0.613, 0.761]	0.706 [0.625, 0.769]	–	–	0.305 [0.239, 0.387]	0.294 [0.231, 0.375]
	Pallidum	0.859 [0.818, 0.889]	0.835 [0.787, 0.871]	–	–	0.141 [0.111, 0.182]	0.165 [0.129, 0.213]
	Putamen	0.859 [0.819, 0.889]	0.874 [0.838, 0.902]	–	–	0.141 [0.111, 0.181]	0.126 [0.098, 0.162]
	Thalamus	0.855 [0.814, 0.887]	0.819 [0.769, 0.857]	–	–	0.145 [0.113, 0.186]	0.181 [0.143, 0.231]
	VNDC	0.700 [0.621, 0.762]	0.726 [0.647, 0.786]	–	–	0.300 [0.238, 0.379]	0.274 [0.214, 0.353]
MD	Brainstem	0.578 [0.469, 0.667]	0.368 [0.234, 0.487]	–	–	0.422 [0.333, 0.531]	0.632 [0.513, 0.766]
	Accumbens	0.608 [0.315, 0.900]	− 0.137 [− 0.521, 0.228]	− 0.037 [− 0.291, 0.200]	0.465 [0.185 ,0.728]	0.429 [0.343, 0.535]	0.672 [0.532, 0.834]
	Amygdala	0.604 [0.511, 0.682]	0.627 [0.528, 0.706]	–	–	0.396 [0.318, 0.489]	0.373 [0.294, 0.472]
	Caudate	0.603 [0.513, 0.678]	0.702 [0.616, 0.769]	–	–	0.397 [0.322, 0.487]	0.298 [0.231, 0.384]
	Hippocampus	0.417 [0.303, 0.518]	0.551 [0.437, 0.646]	–	–	0.583 [0.482, 0.697]	0.449 [0.354, 0.563]
	Pallidum	0.672 [0.596, 0.734]	0.686 [0.593, 0.758]	–	–	0.328 [0.266, 0.404]	0.314 [0.242, 0.407]
	Putamen	0.704 [0.622, 0.768]	0.662 [0.570, 0.735]	–	–	0.296 [0.232, 0.378]	0.338 [0.265, 0.430]
	Thalamus	0.670 [0.573, 0.744]	0.701 [0.616, 0.766]	–	–	0.330 [0.256, 0.427]	0.299 [0.234, 0.384]
	VNDC	0.370 [0.241, 0.484]	0.441 [0.315, 0.548]	–	–	0.630 [0.516, 0.759]	0.559 [0.452, 0.685]
Volume	Brainstem	0.770 [0.637, 0.923]	0.656 [0.520, 0.809]	0.134 [− 0.018, 0.266]	0.225 [0.074, 0.355]	0.096 [0.080, 0.116]	0.119 [0.096, 0.150]
	Accumbens	0.633 [0.569, 0.688]	0.665 [0.595, 0.722]	–	–	0.367 [0.312, 0.431]	0.335 [0.278, 0.405]
	Amygdala	0.731 [0.681, 0.773]	0.727 [0.669, 0.774]	–	–	0.269 [0.227, 0.319]	0.273 [0.226, 0.331]
	Caudate	0.831 [0.688, 0.992]	0.848 [0.701, 1.011]	0.046 [− 0.114, 0.187]	0.041 [− 0.119, 0.184]	0.123 [0.103, 0.147]	0.111 [0.089, 0.140]
	Hippocampus	0.714 [0.553, 0.892]	0.659 [0.483, 0.845]	0.086 [− 0.087, 0.238]	0.127 [− 0.048, 0.283]	0.200 [0.168, 0.239]	0.215 [0.173, 0.268]
	Pallidum	0.616 [0.549, 0.675]	0.717 [0.656, 0.767]	–	–	0.384 [0.325, 0.451]	0.283 [0.233, 0.344]
	Putamen	0.906 [0.875, 0.914]	0.906 [0.885, 0.923]	–	–	0.104 [0.086, 0.125]	0.094 [0.077, 0.115]
	Thalamus	0.771 [0.728, 0.807]	0.771 [0.719, 0.812]	–	–	0.229 [0.193, 0.272]	0.229 [0.193, 0.281]
	VNDC	0.738 [0.561, 0.931]	0.692 [0.504, 0.890]	0.017 [− 0.165, 0.181]	0.072 [− 0.112, 0.238]	0.244 [0.205, 0.293]	0.236 [0.190, 0.295]

Univariate variance component estimates from the correlated factor models are presented. Chi-square difference tests were conducted to assess whether variance components could be dropped without a deterioration of fit. Dashes indicate a dropped variance component

*Accumbens* Nucleus Accumbens, *VNDC* Ventral Diencephalon

### Two-year follow-up results

The contributions to each region and metric are shown in Table 2; Fig. 2 (bottom). The model fit statistics can be found in Supplementary Tables 1–3. Heritability estimates for RNI showed a similar pattern at two-year follow-up, with the pallidum (0.835, 0.787–0.871), putamen (0.874, 0.838–0.902) and thalamus (0.819, 0.767–0.857) again demonstrating the highest heritability. For MD, the amygdala, caudate, pallidum, putamen, and thalamus had similar heritabilities, with estimates in the range of 0.627 to 0.701. As at baseline, structure volumes were most highly heritable for the putamen (0.906, 0.885–0.923) and caudate (0.848, 0.701–1.011).

At two-year follow-up, significant (p < 0.05) contributions of the common environment to MD in the nucleus accumbens (0.465, 0.185–0.728) and the volume of the brainstem (0.225, 0.074–0.355) were observed.

### Longitudinal model results

The correlated factors models assessed the stability of etiological influences on subcortical regions between measures at baseline and the two-year follow-up (Table 3). For RNI, the genetic correlations ranged from 0.743 (amygdala) to 0.970 (caudate). The hippocampus and caudate appear to be genetically stable across time (rA confidence interval includes 1), but the other phenotypes may have novel genetic influences (rA confidence interval do not include 1). The unique environmental correlations ranged from 0.183 (caudate) to 0.660 (amygdala). For MD, the genetic correlations ranged from 0.772 (ventral diencephalon) to 0.967 (amygdala). The confidence intervals for the brainstem, amygdala, hippocampus, pallidum, ventral diencephalon include 1.00, suggesting genetic stability across time. The rA and rC coefficients could not be estimated for the nucleus accumbens MD measure as there was not both a positive A or positive C variance component to correlate. The unique environmental correlations were − 0.038 (nucleus accumbens) to 0.527 (hippocampus). For volume, the genetic correlations were 0.865 (pallidum) to 1 (brainstem). The confidence intervals for the brainstem, nucleus accumbens, caudate, hippocampus, and ventral diencephalon included 1. The unique environmental correlations for the volumetric measures ranged from 0.292 (pallidum) to 0.610 (hippocampus).

**Table 3:** Correlations from the correlated factors model

Measure	Region	*r*P	*r*A	*r*C	*r*E
RNI	Brainstem	**0.724** [0.683, 0.759]	**0.895** [0.828, 0.962]	–	**0.386** [0.243, 0.513]
	Accumbens	**0.706** [0.664, 0.743]	**0.875** [0.810, 0.938]	–	**0.246** [0.079, 0.401]
	Amygdala	**0.708** [0.664, 0.745]	**0.743** [0.659, 0.816]	–	**0.660** [0.544, 0.748]
	Caudate^a^	**0.733** [0.695, 0.767]	**0.970** [0.906, 1.037]	–	**0.183** [0.029, 0.332]
	Hippocampus	**0.761** [0.725, 0.793]	**0.945** [0.887, 1.002]	–	**0.332** [0.178, 0.470]
	Pallidum	**0.844** [0.820, 0.865]	**0.928** [0.898, 0.957]	–	**0.379** [0.229, 0.512]
	Putamen	**0.823** [0.795, 0.846]	**0.904** [0.871, 0.934]	–	**0.295** [0.133, 0.443]
	Thalamus	**0.849** [0.826, 0.869]	**0.958** [0.929, 0.976]	–	**0.293** [0.138, 0.436]
	VNDC	**0.672** [0.625, 0.714]	**0.780** [0.706, 0.848]	–	**0.407** [0.251, 0.541]
MD	Brainstem	**0.534** [0.475, 0.589]	**0.942** [0.783, 1.138]	–	**0.194** [0.040, 0.339]
	Accumbens^b^	**0.493** [0.430, 0.551]	NA	NA	− 0.038 [-0.210, 0.142]
	Amygdala	**0.720** [0.680, 0.755]	**0.967** [0.891, 1.045]	–	**0.327** [0.183, 0.459]
	Caudate	**0.650** [0.601, 0.694]	**0.792** [0.703, 0.875]	–	**0.392** [0.233, 0.529]
	Hippocampus	**0.721** [0.681, 0.756]	**0.941** [0.837, 1.050]	–	**0.527** [0.408, 0.629]
	Pallidum	**0.688** [0.645, 0.727]	**0.949** [0.876, 1.026]	–	0.137 [-0.016, 0.287]
	Putamen	**0.649** [0.600, 0.693]	**0.899** [0.818, 0.982]	–	0.111 [-0.062, 0.278]
	Thalamus	**0.697** [0.653, 0.735]	**0.870** [0.791, 0.951]	–	**0.319** [0.150, 0.472]
	VNDC	**0.341** [0.268, 0.409]	**0.772** [0.546, 1.033]	–	0.049 [-0.102, 0.198]
Volume	Brainstem	**0.899** [0.888, 0.908]	**1.044** [0.999, 1.097]	0.677 [0.303, 0.805]	**0.371** [0.235, 0.489]
	Accumbens	**0.743** [0.714, 0.769]	**0.966** [0.917, 1.016]	–	**0.331** [0.221, 0.434]
	Amygdala	**0.799** [0.775, 0.820]	**0.950** [0.917, 0.982]	–	**0.392** [0.287, 0.487]
	Caudate	**0.921** [0.911, 0.930]	**1.012** [0.983, 1.044]	–	**0.536** [0.436, 0.624]
	Hippocampus	**0.884** [0.870, 0.897]	**1.019** [0.964, 1.085]	–	**0.610** [0.512, 0.690]
	Pallidum	**0.672** [0.635, 0.705]	**0.865** [0.802, 0.928]	–	**0.292** [0.177, 0.400]
	Putamen	**0.902** [0.889, 0.913]	**0.956** [0.942, 0.968]	–	**0.411** [0.302, 0.509]
	Thalamus	**0.773** [0.746, 0.797]	**0.910** [0.874, 0.946]	–	**0.311** [0.194, 0.420]
	VNDC	**0.773** [0.746, 0.797]	**1.036** [0.926, 1.175]	–	**0.294** [0.154, 0.422]

rA, rC, and rE estimates from Correlated Factor models are presented. The etiological correlations are between the baseline and 2-year follow-up measurements of the same phenotype. Dashes indicate non-estimated correlations due to dropped or non-significant variance components. Bolded font indicates p < 0.05, determined with chi-square difference tests. Estimates greater than 1.00 should be interpreted as 1.00

*RNI*restricted normalized isotropic measures.* MD*mean diffusivity measures.* rP*phenotypic correlation.* rA*genetic correlation.* rC*shared environment correlation.* rE*unique environment correlation

^a^The AE model fit slightly worse than the ACE model (Δχ2 = 7.958, p = 0.047), but seemed preferable given that the C variance components were non-significant and rC was inestimable

^b^The rA and rC correlations could not be estimated in the ACE model as there were not both positive A or positive C variance components to correlate

### Stability of measures and heritability estimates

**Fig. 3:**
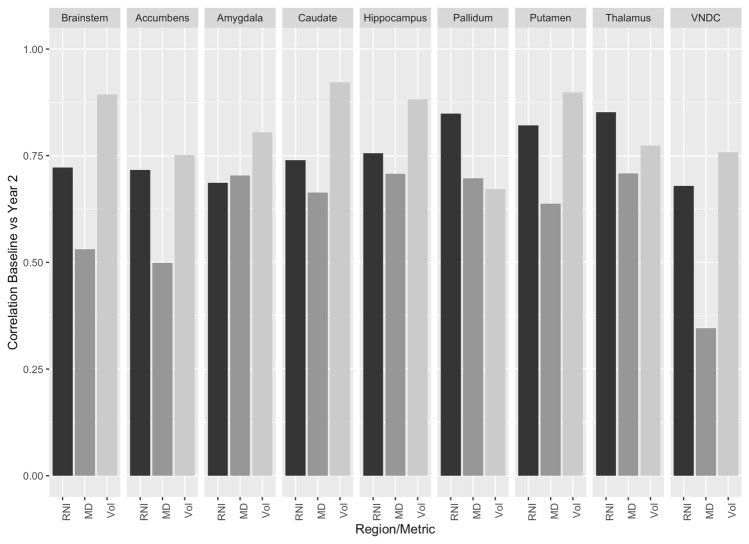
Pearson correlations between RSI, DTI and volumetric measures derived at baseline and two-year follow-up

The Pearson correlations between measures at baseline and two-year follow-up are shown in Fig. 3. For RNI, the correlations ranged from 0.717 (amygdala) to 0.879 (pallidum). For MD, the correlations were substantially lower, ranging from 0.388 (ventral diencephalon) to 0.751 (pallidum). The volume of the pallidum displayed the lowest correlation between time points (0.647), while the caudate was highest (0.931).

**Fig. 4:**
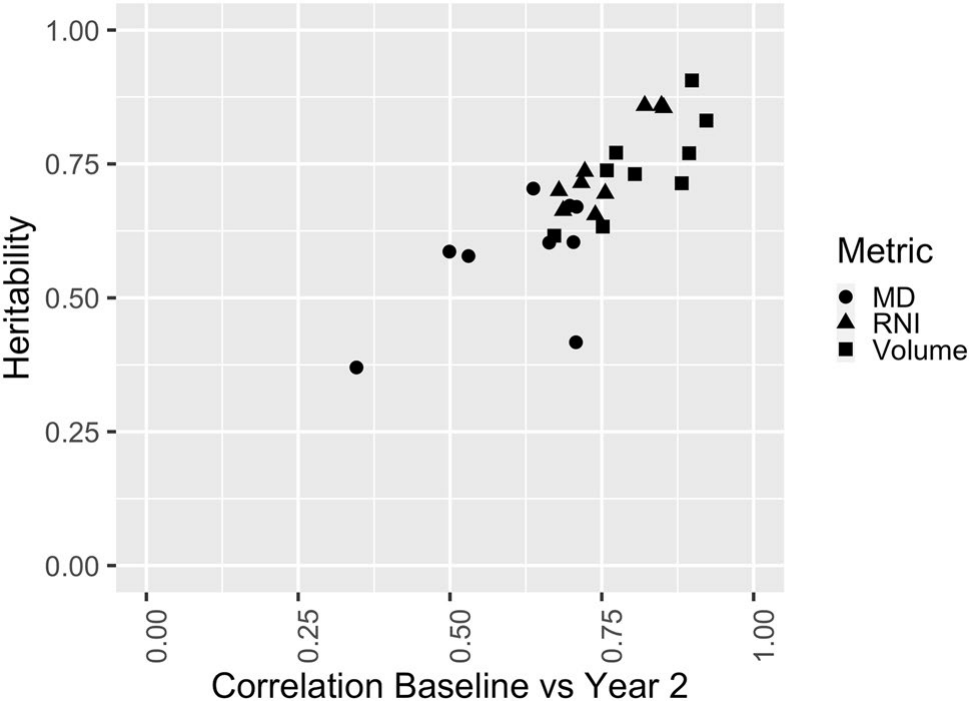
Relationship between heritability determined at baseline and the stability of each metric between baseline and two-year follow-up

Figure 4 shows that those measures that were stable between time points (r = 0.821–0.922, pallidum, putamen, thalamus for RNI, and brainstem, caudate, hippocampus and putamen for volume) demonstrated high heritability estimates (0.714–0.906 at baseline; 0.659–0.906 at two-year follow-up). Conversely, those measures that were least stable showed relatively low heritabilities (such as MD in the brain stem, nucleus accumbens, and ventral diencephalon). The Pearson correlation between heritability and stability was 0.800 (95% CI 0.604–0.905), suggesting that 64% on the variance in heritability across regions/metrics can be explained by the stability of the measurement.

## Discussion

The RNI metric based on an RSI model was more consistent between time points and generally yielded higher heritability estimates compared to MD derived from a diffusion tensor model. The RSI model is biologically based, and substantially more sophisticated than the DTI model, modelling the restricted, hindered, isotropic, anisotropic, and free fluid components separately. By comparison the DTI model combines these components, and the derived MD values are susceptible to contamination, for example from partial volume averaging of free fluid (which has a very high value of MD) with adjacent tissue due to poor tissue segmentation. RNI may be a more reliable microstructure measure to be used in the scope of twin research. A previous study (den Braber et al. 2013) noted poor 5-year retest reliability in the volumes of the nucleus accumbens and pallidum.

The highest microstructure heritability estimates were for the pallidum, putamen, and thalamus. To our knowledge, this is the first study to examine the etiology of subcortical microstructure in adolescents. A prior study (Gillespie et al. 2017) considered heritability of both subcortical MD and volume in middle-aged males. Their results for MD are similar to the present study, with some structures showing higher heritability in adults (caudate, pallidum, putamen), and some lower (amygdala, thalamus). Increasing heritability with age may represent the evolving influence of genetics over development, while decreasing may be due to diverging environmental impacts, especially during adulthood.

For volumes, the highest heritability estimates were for the brainstem, caudate, and putamen. The heritability of subcortical volumes were generally higher or comparable in our cohort compared to prior studies (Swagerman et al. 2014; Yoon et al. 2011). Our results are similar to those of den Braber (den Braber et al. 2013), who found the highest heritability in the left (0.88) and right (0.86) caudate in a cohort of young adults.

The consistency of high heritability estimates across RNI microstructure and volume measures provides evidence that individual variation in these subcortical measures is highly influenced by additive genetic effects. Anomalies in adolescent subcortical structure have been associated with negative outcomes, such as psychopathology (Gurholt et al. 2022; Guyer 2020). The high heritability of these phenotypes suggests they could serve as intermediary phenotypes to identify risk genes for heritable psychopathologies as well as provide insight into possible mechanisms (Glahn et al. 2007; Gurholt et al. 2022).

There was evidence of shared environmental effects influencing brainstem volume and mean diffusivity of the nucleus accumbens at follow-up year 2. However, the nucleus accumbens is often susceptible to low test-retest reliability due to its size and location and this finding should be approached with caution. The brainstem has been associated with neurodevelopmental disorders (Dadalko and Travers 2018). Future research could explore whether shared environmental risk factors, such as nutrition, share variance with the brainstem at this developmentally-sensitive age.

The hippocampus and caudate showed genetic stability (rA confidence intervals included 1) across all three imaging modalities. The putamen and thalamus displayed the possibility of novel genetic influences in all three modalities; however, given the magnitude of the genetic correlations, the genetic effects are mostly stable. All the other phenotypes demonstrated high genetic correlations but were inconsistent across modality on whether there were novel genetic influences. The putamen and thalamus displayed the possibility of novel genetic influences in all three modalities; however, given the magnitude of the genetic correlations, the genetic effects are mostly stable. All the other phenotypes demonstrated high genetic correlations but were inconsistent across modality on whether there were novel genetic influences.

The large genetic correlations across time are suggestive that genetic influences on subcortical development remain relatively stable from ages 10 to 12. Besides the hippocampus and caudate, most of the genetic correlations were less than one in at least one of the imaging modalities, suggesting some novel genetic influences emerging. These findings contradict one previous longitudinal twin study which found no new genetic effects on subcortical brain volumes from ages 9 to 12 (Swagerman et al. 2014); however, the current study had a larger sample size.

In addition to high genetic stability, the subcortical regions displayed unique environmental correlations across time, which were strongest in magnitude for the hippocampus, amygdala, and caudate. Unique environmental correlations can be indicative of a unique risk factor that is not explained by familial influences, such as a traumatic event. Environmental stressors in childhood have been implicated in atypical, sub-nuclei development (McCrory et al. 2012). The literature is especially abundant on structural alterations in the amygdala and hippocampus due to childhood adversity (Calem et al. 2017) and the possible exacerbating impacts these anomalies may have on later psychopathology (McCrory et al. 2012). The unique environmental correlations support that there may be events not captured by familial influence which impact sub-cortical development, and that not all E on subcortical structures is random measurement error. Future research can explore etiological correlations between these subcortical measures in relation to risk factors with psychopathology, such as childhood adversity.

One previous study found that the longitudinal change rate of childhood thalamus, pallidum, and amygdala volumes had a significant heritable component (Brouwer et al. 2017). Future research could explore the heritability of the change rate of subcortical microstructure.

### Limitations

The sample size is an important limitation of twin studies that involve imaging, and this study is no exception. The uncertainties in the derived heritability estimates are large, especially for those regions with moderate or low heritability, perhaps limited by measurement uncertainty. Particularly at baseline (ages 9–10), subject motion can also be problematic, although the ABCD study benefits from the use of prospective motion correction for the structural data acquisition, combined with recommended imaging quality control criteria.

Measurement error is expected to reduce the correlations between MZ twins and DZ twins similarly, resulting in lower heritability estimates for metrics that have low test-retest reliability (Fig. 4, r = 0.8). This implies that the estimates of heritabilities presented may represent a lower bound on the true heritability.

While we controlled for the effect of sex, we did not specifically investigate it. Prior studies have postulated that there are no sex differences in subcortical volumes (den Braber et al. 2013; Vink et al. 2012) which may suggest that the same genes influence the same phenotype in both sexes. However, these studies concentrated on adults.

## Conclusions

Volume and diffusion MRI metrics demonstrated substantial heritability across all subcortical regions in this sample both at baseline and two-year follow-up. The RNI component of diffusion derived from the RSI model was found to be more heritable than MD, perhaps due to its better test-retest reliability. Particularly high heritability of greater than 0.8 was found using the RSI model in the pallidum, putamen, and thalamus, and in volumes of the caudate and putamen. These findings were consistent across time points. The subcortical microstructures and volumes were largely genetically stable, with some small, novel genetic influences. Almost all of the phenotypes had cross-time unique environmental correlations, suggesting that a non-familial risk factor may impact subcortical development.

## Supplementary Information

Below is the link to the electronic supplementary material.

Supplementary material 1 (DOCX 37.0 kb)

## Data Availability

The ABCD data repository grows and changes over time. The ABCD data used in this report came from Curated Data Release 4.0, 10.15154/1523041.

## Code availability (software application or custom code)

The direct variance ACE model used in this work was based on twoACEvc.R, available at. https://hermine-maes.squarespace.com/two.
