# Supplementary material for: Genetic and Environmental Contributions to Subcortical Gray Matter Microstructure and Volume in the Developing Brain
Source: Behav Genet. 2023 Apr 26;53(3):208–18. doi: 10.1007/s10519-023-10142-1 (PMC10154259; doi:10.1007/s10519-023-10142-1)
Supplement: Supplementary file 1 — Supplementary material 1 (DOCX 37.0 kb) [file 10519_2023_10142_MOESM1_ESM.docx]

**Supplementary Table 1**. Model fit statistics of the restriction spectrum imaging measure correlated factor models

|  | Nested Models | -2log-likelihood | df | Δχ^2t^ | df | *p* | CFI | RMSEA |
| --- | --- | --- | --- | --- | --- | --- | --- | --- |
| Brainstem |  | 4376.6 | 1609 | 26.23 | 20 | 0.158 | 0.99 | 0.02 |
|  | Without *r*A | 4518.1 | 1610 | 141.49 | 1 | 1.25e-32 |  |  |
|  | Without *r*E | 4402.2 | 1610 | 25.60 | 1 | 4.19e-07 |  |  |
| Nucleus |  | 5134.6 | 1609 | 17.56 | 20 | 0.616 | 1.00 | 0.00 |
| Accumbens | Without *r*A | 5302.8 | 1610 | 168.21 | 1 | 1.82e-38 |  |  |
|  | Without *r*E | 5142.9 | 1610 | 8.29 | 1 | 3.99e-03 |  |  |
| Amygdala |  | 4573.3 | 1609 | 27.18 | 20 | 0.130 | 0.99 | 0.02 |
|  | Without *r*A | 4651.4 | 1610 | 78.13 | 1 | 9.63e-19 |  |  |
|  | Without *r*E | 4639.8 | 1610 | 66.52 | 1 | 3.47e-16 |  |  |
| Caudate |  | 4364.5 | 1609 | 22.57 | 20 | 0.310 | 1.00 | 0.00 |
|  | Without *r*A | 4558.1 | 1610 | 193.649 | 1 | 5.10e-44 |  |  |
|  | Without *r*E | 4369.9 | 1610 | 5.39 | 1 | 2.02e-02 |  |  |
| Hippocampus |  | 3937.2 | 1609 | 20.94 | 20 | 0.400 | 1.00 | 0.01 |
|  | Without *r*A | 4102.1 | 1610 | 164.88 | 1 | 9.70e-38 |  |  |
|  | Without rE | 3954.1 | 1610 | 16.89 | 1 | 3.96e-05 |  |  |
| Pallidum |  | 6160.7 | 1609 | 18.39 | 20 | 0.562 | 1.00 | 0.00 |
|  | Without *r*A | 6451.5 | 1610 | 290.81 | 1 | 3.32e-65 |  |  |
|  | Without *r*E | 6183.3 | 1610 | 22.57 | 1 | 2.02e-06 |  |  |
| Putamen |  | 3854.7 | 1609 | 15.72 | 20 | 0.734 | 1.00 | 0.00 |
|  | Without *r*A | 4180.5 | 1610 | 325.73 | 1 | 8.17e-73 |  |  |
|  | Without *r*E | 3867.0 | 1610 | 12.30 | 1 | 4.53e-04 |  |  |
| Thalamus |  | 4480.0 | 1609 | 16.44 | 20 | 0.689 | 1.00 | 0.00 |
|  | Without *r*A | 4766.8 | 1610 | 286.80 | 1 | 2.48e-64 |  |  |
|  | Without *r*E | 4493.5 | 1610 | 13.50 | 1 | 2.38e-04 |  |  |
| Ventral |  | 4603.0 | 1609 | 18.08 | 20 | 0.582 | 1.00 | 0.00 |
| Diencephalon | Without *r*A | 4733.6 | 1610 | 130.60 | 1 | 3.03e-30 |  |  |
|  | Without *r*E | 4626.2 | 1610 | 23.25 | 1 | 1.42e-06 |  |  |

*Note.* This table presents the model fit statistics for the ACE bivariate twin models as well as the chi-square difference test for the nested models that drop *r*A, *r*C, or *r*E. df = degrees of freedom. The analyses were limited to the restricted normalized isotropic component.

^t^ The first line for each measure depicts the chi-square for the full model (comparing the -2 log-likelihood to a saturated model). The subsequent rows for each measure correspond to change in chi-square for the reduced model compared to the full model fit.

**Supplementary Table 2**. Model fit statistics of the mean diffusivity measures correlated factor models

|  | Nested models | -2log-likelihood | df | Δχ^2t^ | df | *p* | CFI | RMSEA |
| --- | --- | --- | --- | --- | --- | --- | --- | --- |
| Brainstem |  | 308.0 | 1609 | 21.38 | 20 | 0.375 | 1.00 | 0.01 |
|  | Without *r*A | 374.1 | 1610 | 66.05 | 1 | 4.39e-16 |  |  |
|  | Without *r*E | 314.2 | 1610 | 6.12 | 1 | 0.0134 |  |  |
| Nucleus |  | 6882.8 | 1606 | 18.20 | 17 | 0.376 | 1.00 | 0.01 |
| Accumbens | Without *r*A | 6898.3 | 1606 | 15.46 | 1 | 8.41e-05 |  |  |
|  | Without *r*C | 6882.8 | 1606 | 7.21e-04 | 1 | 0.979 |  |  |
|  | Without *r*E | 6883.0 | 1607 | 0.17 | 1 | 0.676 |  |  |
| Amygdala |  | 6898.9 | 1609 | 33.49 | 20 | 0.0298 | 0.98 | 0.03 |
|  | Without *r*A | 7030.5 | 1610 | 131.64 | 1 | 1.79e-30 |  |  |
|  | Without *r*E | 6917.5 | 1610 | 18.64 | 1 | 1.58e-05 |  |  |
| Caudate |  | 7604.4 | 1609 | 47.37 | 20 | 5.22e-4 | 0.95 | 0.04 |
|  | Without *r*A | 7706.5 | 1610 | 102.12 | 1 | 5.23e-24 |  |  |
|  | Without *r*E | 7625.4 | 1610 | 21.00 | 1 | 4.59e-06 |  |  |
| Hippocampus |  | 7335.4 | 1609 | 31.21 | 20 | 0.0525 | 0.98 | 0.03 |
|  | Without *r*A | 7401.0 | 1610 | 65.64 | 1 | 5.42e-16 |  |  |
|  | Without *r*E | 7393.2 | 1610 | 57.77 | 1 | 2.94e-14 |  |  |
| Pallidum |  | 5800.2 | 1609 | 38.91 | 20 | 0.00683 | 0.97 | 0.04 |
|  | Without *r*A | 5969.0 | 1610 | 168.72 | 1 | 1.41e-38 |  |  |
|  | Without *r*E | 5803.3 | 1610 | 3.089 | 1 | 7.88e-02 |  |  |
| Putamen |  | 4196.1 | 1609 | 36.73 | 20 | 0.0126 | 0.96 | 0.03 |
|  | Without *r*A | 4339.8 | 1610 | 143.79 | 1 | 3.95e-33 |  |  |
|  | Without *r*E | 4197.6 | 1610 | 1.58 | 1 | 0.208 |  |  |
| Thalamus |  | 5583.2 | 1609 | 38.92 | 20 | 0.00682 | 0.97 | 0.04 |
|  | Without *r*A | 5709.1 | 1610 | 125.93 | 1 | 3.19e-29 |  |  |
|  | Without *r*E | 5596.4 | 1610 | 13.23 | 1 | 2.76e-04 |  |  |
| Ventral |  | 7255.4 | 1609 | 19.26 | 20 | 0.505 | 1.00 | 0.00 |
| Diencephalon | Without *r*A | 7293.0 | 1610 | 35.50 | 1 | 2.55e-09 |  |  |
|  | Without *r*E | 7257.9 | 1610 | 0.40 | 1 | 0.525 |  |  |

*Note.* This table presents the model fit statistics for the ACE bivariate twin models as well as the chi-square difference test for the nested models that drop *r*A, *r*C, or *r*E. df = degrees of freedom.

^t^ The first line for each measure depicts the chi-square for the full model (comparing the -2 log-likelihood to a saturated model). The subsequent rows for each measure correspond to change in chi-square for the reduced model compared to the full model fit.

**Supplementary Table 3**. Model fit statistics of the volumetric measure correlated factor models

|  | Nested models | -2log-likelihood | df | Δχ^2t^ | df | *p* | CFI | RMSEA |
| --- | --- | --- | --- | --- | --- | --- | --- | --- |
| Brainstem |  | 15503.7 | 2427 | 70.47 | 17 | 1.79e-08 | 0.98 | 0.06 |
|  | Without *r*A | 15700.3 | 2428 | 196.55 | 1 | 1.18e-44 |  |  |
|  | Without *r*C | 15506.4 | 2428 | 2.64 | 1 | 1.04e-01 |  |  |
|  | Without *r*E | 15529.6 | 2428 | 25.89 | 1 | 3.61e-07 |  |  |
| Nucleus |  | 2582.4 | 2427 | 32.08 | 20 | 0.0425 | 0.99 | 0.03 |
| Accumbens | Without *r*A | 2817.4 | 2428 | 234.95 | 1 | 4.97e-53 |  |  |
|  | Without *r*E | 2615.2 | 2428 | 32.80 | 1 | 1.02e-08 |  |  |
| Amygdala |  | 6050.8 | 2430 | 18.46 | 20 | 0.557 | 1.00 | 0.00 |
|  | Without *r*A | 6356.2 | 2431 | 305.40 | 1 | 2.20e-68 |  |  |
|  | Without *r*E | 6099.3 | 2431 | 48.56 | 1 | 3.20e-12 |  |  |
| Caudate |  | 8855.3 | 2427 | 49.41 | 17 | 5.20e-05 | 0.99 | 0.05 |
|  | Without *r*A | 9052.5 | 2428 | 197.17 | 1 | 8.68e-45 |  |  |
|  | Without *r*C | 8855.4 | 2428 | 0.0153 | 1 | 0.901 |  |  |
|  | Without *r*E | 8937.5 | 2428 | 82.15 | 1 | 1.26e-19 |  |  |
| Hippocampus |  | 8356.8 | 2427 | 39.84 | 17 | 0.00137 | 0.99 | 0.04 |
|  | Without *r*A | 8443.3 | 2428 | 86.48 | 1 | 1.41e-20 |  |  |
|  | Without *r*C | 8357.34 | 2428 | 0.559 | 1 | 4.55e-01 |  |  |
|  | Without *r*E | 8457.5 | 2428 | 100.65 | 1 | 1.10e-23 |  |  |
| Pallidum |  | 6458.2 | 2430 | 47.94 | 20 | 4.34e-4 | 0.97 | 0.04 |
|  | Without *r*A | 6684.5 | 2431 | 226.31 | 1 | 3.81e-51 |  |  |
|  | Without *r*E | 6482.1 | 2431 | 23.85 | 1 | 1.04e-06 |  |  |
| Putamen |  | 21276.2 | 2430 | 59.92 | 20 | 7.34e-06 | 0.98 | 0.05 |
|  | Without *r*A | 21889.0 | 2431 | 612.89 | 1 | 2.63e-135 |  |  |
|  | Without *r*E | 21324.7 | 2431 | 48.57 | 1 | 3.19e-12 |  |  |
| Thalamus |  | 11210.6 | 2430 | 51.18 | 20 | 1.50e-4 | 0.98 | 0.05 |
|  | Without *r*A | 11566.2 | 2431 | 355.59 | 1 | 2.57e-79 |  |  |
|  | Without *r*E | 11236.1 | 2431 | 25.43 | 1 | 4.58e-07 |  |  |
| Ventral |  | 8149.9 | 2427 | 69.04 | 17 | 3.16e-08 | 0.96 | 0.06 |
| Diencephalon | Without *r*A | 8244.0 | 2428 | 94.06 | 1 | 3.06e-22 |  |  |
|  | Without *r*C | 8150.2 | 2428 | 0.245 | 1 | 0.621 |  |  |
|  | Without *r*E | 8166.6 | 2428 | 16.71 | 1 | 4.345e-05 |  |  |

*Note.* This table presents the model fit statistics for the ACE bivariate twin models as well as the chi-square difference test for the nested models that drop *r*A, *r*C, or *r*E. df = degrees of freedom.

^t^ The first line for each measure depicts the chi-square for the full model (comparing the -2 log-likelihood to a saturated model). The subsequent rows for each measure correspond to change in chi-square for the reduced model compared to the full model fit.
